# Novel Dual Tracer Indocyanine Green and Radioisotope Versus Gold Standard Sentinel Lymph Node Biopsy in Breast Cancer: The GREENORBLUE Trial

**DOI:** 10.1245/s10434-023-13824-6

**Published:** 2023-07-04

**Authors:** Chu Luan Nguyen, Michael Zhou, Neshanth Easwaralingam, Jue Li Seah, Farhad Azimi, Cindy Mak, Carlo Pulitano, Sanjay Warrier

**Affiliations:** 1 Department of Breast Surgery, Chris O’Brien Lifehouse, Camperdown, NSW Australia; 2 Department of Surgery, Royal Prince Alfred Hospital, Camperdown, NSW Australia; 3 Department of Surgery, The University of Sydney, Camperdown, NSW Australia

## Abstract

**Background:**

The methods for sentinel lymph node (SLN) biopsy in breast cancer have been variable in type and number of tracers. Some units have abandoned the use of blue dye (BD) due to adverse reactions. Fluorescence-guided biopsy with indocyanine green (ICG) is a relatively novel technique. This study compared the clinical efficacy and costs between novel dual tracer ICG and radioisotope (ICG-RI) with “gold standard” BD and radioisotope (BD-RI).

**Methods:**

Single-surgeon study of 150 prospective patients with early breast cancer undergoing SLN biopsy (2021-2022) using ICG-RI compared with a retrospective cohort of 150 consecutive previous patients using BD-RI. Number of SLNs identified, rate of failed mapping, identification of metastatic SLNs, and adverse reactions were compared between techniques. Cost-minimisation analysis performed by using Medicare item numbers and micro-costing analysis.

**Results:**

Total number of SLNs identified with ICG-RI and BD-RI was 351 and 315, respectively. Mean number of SLNs identified with ICG-RI and BD-RI was 2.3 (standard deviation [SD] 1.4) and 2.1 (SD 1.1), respectively (*p* = 0.156). There were no cases of failed mapping with either dual technique. Metastatic SLNs were identified in 38 (25.3%) ICG-RI patients compared with 30 (20%) BD-RI patients (*p* = 0.641). There were no adverse reactions to ICG, whereas four cases of skin tattooing and anaphylaxis were associated with BD (*p* = 0.131). ICG-RI cost an additional AU$197.38 per case in addition to the initial cost for the imaging system. Clinical trial registration: ACTRN12621001033831.

**Conclusions:**

Novel tracer combination, ICG-RI, provided an effective and safe alternative to “gold standard” dual tracer. The caveat was the significantly greater costs associated with ICG.

**Supplementary Information:**

The online version contains supplementary material available at 10.1245/s10434-023-13824-6.

“Gold standard” lymphatic mapping for sentinel lymph node (SLN) biopsy involves the combination of blue dye (BD; patent blue, methylene blue, or isosulphan blue) and radioisotope (RI) labelled with technetium-99m.^1,2^ Use of BD and RI for lymphatic mapping in breast cancer were first described in the early 1990s. The combination of BD-RI has been used as a standard technique following this with high SLN detection rates and low false-negative rates of 96.7% and 5.5%, respectively.^3–8^

Methods for nodal identification have been variable in terms of type and number of tracers used since the advent of SLN biopsy as the principal procedure for axilla staging in breast cancer. Use of BD has been widespread since its introduction nearly three decades ago; some units even use BD alone. Some units, however, have abandoned BD from combination with RI due to potential anaphylaxis.^9,10^

Indocyanine green (ICG) fluorescence imaging utilises the green fluorophore dye, ICG, with a near-infrared camera to visualise subcutaneous lymphatic flow in real-time for axillary dissection of SLNs.^11–13^ The dye binds to plasma albumin and acts as a fluorescent tracer of lymphatic channels and nodes seen on a near-infrared display.^14^ It has been shown in comparative cohort studies to be equivalent to single technique using RI and superior to single technique with BD.^15^

For a new technology to be adopted into clinical practice, it should ideally be as effective as the current “gold standard” and at a reasonable cost. This study compared the clinical efficacy and costs between novel ICG-RI and “gold standard” BD-RI for SLN biopsy in early breast cancer in terms of number of SLNs identified, rate of failed mapping, identification of metastatic SLNs, and adverse reactions to dye tracer.

## Methods

### Study Design and Participants

This was a single-centre, single surgeon, study of prospective patients with early breast cancer undergoing SLN biopsy using ICG-RI from April 2021 to October 2022. It was registered on the Australian New Zealand Clinical Trials Registry (ANZCTR: ACTRN12621001033831) and followed the Standards for Reporting of Diagnostic Accuracy (STARD) and Consolidated Health Economic Evaluation Reporting Standards 2022 (CHEERS 2022) guidelines.^16^

Inclusion criteria was female, age ≥18 years, with early breast cancer confirmed by core or fine-needle biopsy, a clinically node-negative axilla, and scheduled for SLN biopsy with breast conserving surgery (BCS). Exclusion criteria was patients with clinically positive lymph nodes, previous axillary surgery or neoadjuvant therapy, or known contraindication to ICG. Patients who met inclusion criteria underwent SLN biopsy with dual-tracer ICG-RI technique. This cohort was compared with a retrospective cohort of consecutive patients, meeting the same inclusion criteria (except for ICG contraindications), who underwent SLN biopsy using standard BD-RI before the introduction of ICG.

### Surgical Technique

Eligible patients underwent preoperative lymphoscintigraphy with subdermal/peritumour injection of 15-20 MBq of technetium-99m the day before surgery. At the time of surgery after anaesthesia and immediately before the operation, 1 mL of Infracyanine^®^ 25 mg/10 mL (SERB, Paris, France) was injected subdermally into the peri-areolar area. Movement of ICG in the lymphatic ducts was facilitated by manual massage.

Fluorescence of ICG was elicited and detected by the near-infrared camera (SPY-PHI, STRYKER, Sydney, Australia). ICG generates fluorescence by contacting plasma proteins in the lymphovascular system. It absorbs near-infrared light and emits a fluorescent signal when particles return from an excited to ground state.^14^ Lymphatic drainage was visualised in real-time on a monitor and the fluorescence followed from injection site towards the axilla. SLN mapping and biopsy proceeded through either a BCS or axillary incision.

Assessment of the surgical field using the near-infrared camera was applied continuously throughout the surgical exploration. Fluorescent lymphatic channels were dissected and followed to the first ICG avid lymph node. Fluorescent lymph nodes (“ICG-positive”) were then localised and excised. Excised ICG-positive nodes were then tested for radioactivity using the gamma-detecting probe and classified as “hot” (RI-positive”) or “cold” (“RI-negative”). SLN removal continued until no residual fluorescence was visible in the axilla.

Finally, the axilla was inspected with the gamma-detecting probe to determine whether any radioactivity was left. If there was significant residual radioactivity the hot spot was removed and examined. The number of SLNs (“ICG-positive,” “RI-positive,” or both) removed from each patient were documented. This allowed for calculation of nodal detection rates for ICG alone and in combination with RI. A SLN was defined as any node with at least 10% of the hottest nodal counts in regards to radioactivity. All SLNs were sent for histopathology analysis.

Patients in the BD-RI cohort underwent the same preoperative lymphoscintigraphy as the ICG-RI cohort. At the time of surgery after anaesthesia and immediately before the operation, 2.5 mL of Patent Blue V dye (125 mg/5 mL) was injected in the same manner. Identification of SLNs was performed as usual. The “blue” SLNs were excised and sent for histopathology analysis. The number of SLNs was recorded numerically and whether “blue,” “RI-positive,” or both, allowing calculation of nodal detection rates for BD alone and in combination with RI.

### Histopathology Assessment

**Fig. 1:**
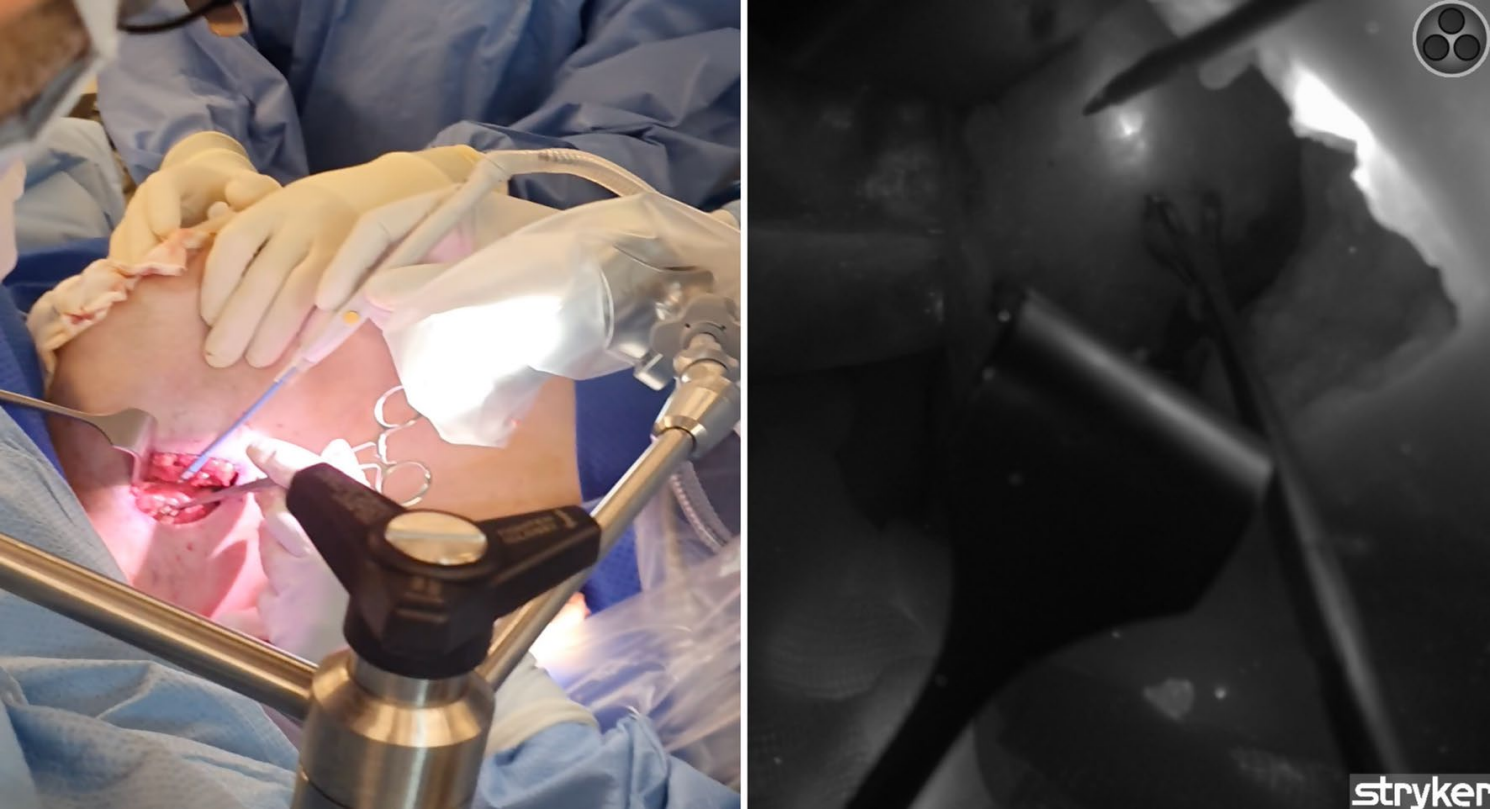
“ICG-positive” sentinel lymph node identified with intraoperative indocyanine green fluorescence elicited on near-infrared camera

Lymph nodes were serially sectioned at intervals and subsequently stained with hematoxylin and eosin (H&E). The pathology report included number of lymph nodes examined, as well as how many were benign, contained isolated tumour cells (<0.2 mm in size), micrometastasis (0.2–2 mm in size) or macrometastasis (>2 mm in size), and extracapsular invasion (Fig. 1).

### Outcomes

Primary outcomes were the number of SLNs identified, rate of failed mapping, identification of metastatic SLNs, and adverse reactions to dye tracer between ICG-RI and BD-RI techniques. Secondary outcomes were costs associated with the two techniques. Demographic information included tumour type, previous axillary surgery or neoadjuvant therapy, and body mass index (BMI). Intraoperative information included the number of nodes identified with each technique and operation duration. Postoperative information included hospital admission duration, complications (adverse reactions and anaphylaxis to tracers), significant complications related to SLN biopsy (Clavien-Dindo grade ≥III), number of metastatic lymph nodes on histopathology, and follow-up visit information up to 90 days postoperative. Anaphylaxis was defined as a severe multisystemic reaction based on the National Institute of Health.^17^

### Cost Analysis

Cost minimisation analysis was utilised as outcomes of the alternative technique being compared were assumed to be equivalent.^18^ The perspective of a third-party payer (Medicare) was adopted. The fixed cost was the purchase price of the SPY-PHI fluorescence imaging system. There was no fixed cost for BD, because there was no initial outlay cost. The variable cost was the cost associated with each use of ICG or BD drug. Theatre costs were based on micro-costing including costs for personnel and consumables related to ICG fluorescence technique.

### Sample-size Calculation

The BD-RI technique was treated as “gold standard” with established evidence of capturing at least 97% of the SLNs. At least 260 SLNs excised were calculated to demonstrate equivalence between the two dual techniques with a 5% margin, 5% alpha error, and 80% power (Supplemental material).^19^ At least 18 months was estimated to be needed to reach accrual target for each technique based on the institution’s most recent accrual rates and accounting for 10% attrition rates.

### Statistical Analysis

Continuous variables were presented as means with standard deviation (SD) or as median with interquartile ranges (IQR) and dichotomous and categorical data as frequencies with percentages (Supplemental material). The *t* test was used to compare the mean for continuous variables between the two groups. Chi-squared test was used to compare the proportions of categorical variables between the two groups. Results with *p* ≤ 0.05 were considered to be statistically significant. Statistical analysis was performed with RStudio, v2022.07.2.

## Results

### Demographics

One hundred fifty patients with early breast cancer underwent SLN biopsy using ICG-RI. This was compared with a cohort of 150 patients who underwent SLN biopsy using BD-RI. There were no significant differences between the patients in the ICG-RI cohort compared with the BD-RI cohort with respect to age, BMI, ASA status, and tumour type, and receptor status. The only differences were that the ICG-RI cohort had significantly less tumour grade III cases (16% vs. 29.3%, *p* < 0.001) and somewhat smaller median tumour size (18 vs. 21 mm, *p* = 0.045) compared with the BD-RI cohort (Table 1).

**Table 1:** Demographics of ICG-RI and BD-RI sentinel lymph node biopsy cohorts

Characteristic	ICG-RI, N = 150	BD-RI, N = 150	*p* value
Patient			
Age, yr, median (IQR)	60 (52, 71)	64 (50, 74)	0.51
BMI, kg/m^2^, mean (SD)	25.8 (4.8)	25.6 (4.4)	0.68
ASA, median (IQR)	2 (2, 2)	2 (2, 2)	1
Tumour			
Type, N (%)			0.809
Invasive ductal	112 (74.7)	121 (80.7)	
Invasive lobular	15 (10)	12 (8)	
Ductal carcinoma in situ	9 (6)	7 (4.7)	
Mixed	7 (4.7)	5 (3.3)	
Other^a^	7 (4.7)	5 (3.3)	
Grade, N (%)			**< 0.001**
I	42 (28)	38 (25.3)	
II	71 (47.3)	51 (34)	
III	24 (16)	44 (29.3)	
Size, mm, median (IQR)	18 (12, 28)	21 (14, 32)	**0.045**
Receptor status			
ER+	123 (82)	110 (73.3)	0.295
PR+	116 (77.3)	102 (68)	0.259
HER2+	17 (11.3)	17 (11.3)	0.953
Triple negative	10 (6.7)	15 (10)	0.344
Ki67 index ≥14%	61 (40.7)	70 (46.7)	0.174

*ICG-RI* indocyanine green and radioisotope; *BD-RI* blue dye and radioisotope; *BMI* body mass index; *ASA* American Society of Anesthesiologists Classification; *ER+* estrogen receptor-positive; *PR+* progesterone receptor-positive; *HER2+* herceptin receptor-positive; *IQR* interquartile range; *SD* standard deviation

^a^Includes invasive mucinous carcinoma, micropapillary carcinoma, and solid papillary carcinoma

### Sentinel Lymph Node Detection Rates

There was comparable efficacy between novel dual tracer ICG-RI and “gold standard” BD-RI. Mean number of SLNs identified with ICG-RI and BD-RI was 2.3 (SD 1.4) and 2.1 (SD 1.1), respectively (*p* = 0.156). For the ICG-RI cohort, a total of 351 SLNs containing either one or both tracers were identified. Dual tracer was found in 330 (94%) SLNs. There were no cases of failed mapping with ICG-RI dual technique. Failed mapping rate for ICG alone was 3.4% (12/351) and 2.6% (9/351) for RI alone. For the BD-RI cohort, a total of 315 SLNs containing one or both tracers were identified. Dual tracer was found in 298 (94.6%) SLNs. There were no cases of failed mapping with BD-RI dual technique. Failed mapping rate for BD alone was 2.9% (9/315) and 2.5% (8/315) for RI alone (Table 2).

**Table 2:** Number of sentinel lymph nodes identified with ICG-RI compared to BD-RI

	ICG-RI	BD-RI	*p* value
Dye mapped; RI mapped, N (%)	330 (94)	298 (94.6)	0.156
Dye mapped; RI not mapped,^a^ N (%)	9 (2.6)	8 (2.5)	0.845
Dye not mapped; RI mapped,^b^ N (%)	12 (3.4)	9 (2.9)	0.606
Total SLNs	351	315	

*ICG-RI* indocyanine green and radioisotope; *BD-RI* blue dye and radioisotope

^a^Failed RI mapping

^b^Failed dye mapping

### Metastatic Lymph Nodes

There were no statistically significant differences identified with respect to identification of metastatic SLNs between the two techniques (Table 3). In the ICG-RI cohort, metastatic SLNs were found in 38 of 150 (25.3%) patients. Among these 38 patients, there were 46 metastatic SLNs, which represented 13.1% of all 351 SLNs excised. In the BD-RI cohort, metastatic SLNs were found in 30 of 150 (20%) patients. Among these 30 patients, there were 41 metastatic SLNs, which represented 13% of all 315 SLNs excised (*p* = 0.641; Table 3).

**Table 3:** Number of metastatic sentinel lymph nodes identified with ICG-RI compared with BD-RI

	ICG-RI	BD-RI	*p* value
Dye mapped; RI mapped, N (%)	43 (93.5)	41 (100)	0.847
Dye mapped; RI not mapped,^a^ N (%)	1 (2.2)	0	0.318
Dye not mapped; RI mapped,^b^ N (%)	2 (4.3)	0	0.318
Total SLNs	46	41	

*ICG-RI* indocyanine green and radioisotope; *BD-RI* blue dye and radioisotope

^a^Failed RI mapping

^b^Failed dye mapping

### Adverse Reactions

There were no adverse reactions related to ICG, including allergic reactions, skin necrosis, or staining, identified in the ICG-RI cohort up to 90 days follow-up. Four adverse reactions were attributed to BD in the BD-RI cohort; this was not statistically significant (*p* = 0.131; Table 4). This included two cases of intraoperative anaphylaxis and two cases of skin tattooing. One case of anaphylaxis had hypotension, bronchospasm, and a skin rash, requiring transfer to intensive care for inotropic support. The second case of anaphylaxis had hypoxaemia and a skin rash, which resolved in theatre following corticosteroids. In both cases, serum tryptase level was raised. Skin prick and intradermal tests demonstrated a severe reaction to BD. There were no other significant (Clavien-Dindo grade ≥III) complications related to SLN biopsy, except for the case of anaphylaxis requiring intensive care admission.

**Table 4:** Outcomes of sentinel lymph node biopsy using ICG-RI compared to BD-RI

	ICG-RI	BD-RI	*p* value
Complication,^a^ N (%)	0	1 (.7)	1
Dye adverse reaction, N (%)	0	4 (2.7)^b^	0.131
Operation duration, mean mins (SD)	59 (21.5)	54.6 (20.9)	0.192
Length of stay, mean days (SD)	0.4 (1.1)	0.8 (1.6)	0.171

*ICG-RI* indocyanine green and radioisotope; *BD-RI* blue dye and radioisotope; *SD* standard deviation

^a^Clavien-Dindo grade ≥III complication related to sentinel lymph node biopsy

^b^Two cases of anaphylaxis and two cases of skin tattooing

### Cost Analysis

The fixed cost for ICG use included the fluorescence imaging system (total, AU$88,720; camera, AU$46,570; console, AU$35,150; software, AU$7,000). There was no initial outlay cost for BD use. The ongoing variable costs were costs associated with each vial of ICG drug (AU$124.88), which was greater than each vial of BD drug (AU$80.00). Theatre costs were based on micro-costing, including costs for personnel and consumables related to ICG fluorescence technique. The mean operation duration was longer for ICG-RI compared with BD-RI (59 vs. 54.6 minutes, respectively, *p* = 0.192). Although this was not statistically significant, it was estimated that an additional 5 minutes was needed for each ICG-RI case for the cost-analysis.^20,21^ This resulted in a total additional AU$197.38 for each ICG-RI case in addition to the fixed cost of the imaging system (Table 5).

**Table 5:** Costs of using indocyanine green fluorescence compared to blue dye for sentinel lymph node biopsy

Item	Cost, AU$	
	BD	ICG
Initial outlay cost		
Fluorescence imaging system^a^		88720.00
Drugs		
Patent blue V dye (per vial, 125 mg/5 mL)	80.00	
Indocyanine green drug (per vial, 25 mg/10 mL)		124.88
Theatre consumables		
Near-infrared camera sterile cover		5.80
Theatre costs		
Surgeon/ anaesthetist fee (per min)	3.54	3.54
Other personnel fee^b^ (per min)	10.00	10.00
Theatre room fee (per min)	15.80	15.80
Operation (Medicare)		
Wide local excision	687.30	687.30
Sentinel lymph node biopsy	673.85	673.85
Total cost per case^c^	3054.85	3252.23
Total additional cost of ICG fluorescence per case		197.38

*AU$* Australian dollars; *BD* blue dye; *ICG* indocyanine green

^a^SPY-PHI (STRYKER, Arndell Park, Sydney Australia)

^b^Includes theatre scrub and anaesthetic nursing staff

^c^Cost per median operation duration of 55 minutes for BD-RI and an additional 5 minutes of time added due to ICG-RI; not inclusive of initial outlay cost of imaging system

## Discussion

SLN biopsy with BD-RI has become a diagnostic standard of care in clinically node-negative, early breast cancer within the past two decades.^22,23^ For a new technology to be adopted into clinical practice, it should ideally be as effective as current “gold standard” and at a reasonable cost. This study compared combination of ICG-RI tracer for SLN biopsy in early breast cancer with a control cohort using gold standard BD-RI. This is unique compared to previous studies which used fluorescence-guided SLN biopsy in combination with a comparator tracer (RI and/or BD) in the one cohort.^8,15,24,25^

Blue dye was replaced with ICG in this study after a decade of its use. The outcomes of this transition demonstrate equivalency between the two dual techniques in terms of number of SLNs identified, rate of failed mapping, and identification of metastatic SLNs. The abandonment of BD in favour for ICG has not only been justified in terms of SLN biopsy efficacy, but so far has been safe with no adverse reactions associated with ICG. These results could aid surgeons that use BD in considering an alternative tracer with the caveat being higher costs.

The SLN detection rates were equivalent for both ICG-RI and BD-RI. There were no cases of failed mapping with either dual technique. The overall node positivity rate was similar between the two techniques. There is a paucity of data on this new approach with ICG-RI; one study used this combination in a small series (32 patients) and reported a SLN detection rate of 100%. The study also added BD to the ICG-RI technique and did not compare it to the “gold standard”.^15,26^ The accuracy of ICG fluorescence SLN mapping is partly due to its ability to allow for sequential nodal dissection guided by visualisation of lymphatics. Nodes at a variable depth from the gamma-detecting probe are detected as hot spots irrespective of anatomical lymphatic flow when RI is used. This may make identifying nodes in sequential order more difficult with RI.^27,28^ Combining ICG and RI allows the two modalities to complement each other for SLN localisation. In this study, all SLNs could be detected by both fluorescence imaging and gamma-detecting probe.

There were initial concerns with ICG fluorescence detecting higher nodal counts between three to five per patient in early studies. This is thought to be related to the low molecular weight and fast diffusion of ICG through lymphatics.^8^ A larger number of SLNs sampled may increase risk for upper limb lymphoedema, sensory deficit, and reduced shoulder function. There was a significant difference in morbidity rates when comparing SLN biopsy with axillary dissection in landmark trials (25% vs. 70%, respectively).^23,29^ More recent studies, including this study, have reported average nodal counts of approximately two per patient, which could be due to improvements in near-infrared technology and ICG fluorescence protocols.^30–33^

The importance of BD use as a tracer for SLN biopsy has come under scrutiny since the introduction of RI and more recently, ICG. Skin tattooing and skin or fat necrosis at site of injection are well described.^34,35^ A recent meta-analysis reported that overall incidence of anaphylaxis to BD during SLN biopsy for breast cancer surgery was 0.083% (1/1,200 patients).^35^ Although the relative risk is small, it can be argued that if a safer alternative is available, then it should be considered to improve patient safety. A small number of adverse reactions, including skin tattooing and anaphylaxis, was experienced in this study’s BD-RI cohort. ICG has an excellent safety profile with a low incidence of adverse events (1/42,000 patients).^36–38^ There was, however, no significant difference in safety in relation to adverse reactions to tracers in the authors’ early transition to ICG.

Understanding the learning curve of a new surgical technique is important in considering its adoption. No trials, to date, have evaluated the learning curve of SLN biopsy with ICG. Surgeons require approximately 40 cases for SLN biopsy proficiency as described in the ALMANAC trial using “gold standard,” dual technique.^39^ Use of BD as a single technique has been reported to be more difficult to learn compared with RI. This is thought to be due to auditory cues that RI provides, which allows guidance through tissue. Blue dye only provides feedback after dissection upon direct visualisation of the node.^40^ Both BD and RI are static in their feedback, as they only provide signal where sufficient accumulation of tracer has occurred in nodes. ICG fluorescence provides dynamic real-time visualisation of lymphatic channels as ICG travels from injection site towards the SLN. It has a simple protocol and potentially short learning curve, as it appears to require less navigational skill compared with RI or BD.^41^ The additional time added to the operation with use of ICG-RI, however, could negatively affect efficiency and workflow in the surgical setting.

The costs involved with a new technology compared to current “gold standard” is a key consideration in its adoption. Fluorescence imaging requires additional expenditure, including costs of ICG drug and the dedicated camera. This study found that tracer combination of ICG-RI was more expensive than BD-RI. Although there were significant costs associated with adverse reactions from BD, the initial capital outlay costs of ICG were significantly higher. These costs associated with ICG may not be worthwhile to prevent such a low rate of adverse reactions related to BD. The adoption of ICG-RI may pose financial challenges due to its higher initial outlay costs as well as the recurrent cost of ICG drug. These cost considerations should be taken into account when considering the feasibility of implementing ICG-RI in real-world, clinical practice. Increased uptake of fluorescence technology could potentially lower costs of the imaging system, which is a significant limiting factor in its adoption. ICG fluorescence as a single technique would, in theory, be less expensive than BD-RI. Use of RI has additional costs from hospital infrastructure to accommodate radioactive materials, specialist staff for lymphoscintigraphy, and patient travel.^42^ From a cost perspective alone, ICG is preferable to BD-RI. Use of ICG as a single tracer, however, has yet to be validated.

This study is limited in that it was nonrandomised and lacked a direct comparison to “gold standard” technique, which weakens the strength of the conclusions that can be drawn. No trials to date have compared ICG alone with the “gold standard” dual technique. This is likely due to ICG being a relatively novel technique with the safer option to combine it with another tracer to minimise risk of missing metastatic SLNs. The novel combination of ICG-RI could represent a transition phase with ICG used as a sole tracer in future.^28^ This process would benefit from standardisation of the technique and validation from randomised, controlled trials. This along with an evaluation of the health economics would provide valuable data to assist with potential uptake of this technology.

This study provides data supporting efficacy of novel dual technique, ICG-RI, for SLN biopsy in early breast cancer with comparable detection rates to the “gold standard” dual technique. Widespread abandonment of RI in the near future is unlikely given its well-established data. Using ICG as an adjunct to RI would be a more pragmatic approach to consider. It can be complementary to RI in the absence of BD. Novel tracer combination, ICG-RI, provided an effective and safe alternative to “gold standard” dual tracer. The caveat was the significantly greater costs associated with ICG.

## Supplementary Information

Below is the link to the electronic supplementary material.

Supplementary file 1 (DOCX 624 KB)

## Data Availability

The data that support the findings of this study are available on request from the corresponding author, CLN.
