# Supplementary material for: Novel Dual Tracer Indocyanine Green and Radioisotope Versus Gold Standard Sentinel Lymph Node Biopsy in Breast Cancer: The GREENORBLUE Trial
Source: Ann Surg Oncol. 2023 Jul 4;30(11):6520–7. doi: 10.1245/s10434-023-13824-6 (PMC10507001; doi:10.1245/s10434-023-13824-6)
Supplement: Supplementary file 1 — (DOCX 624 KB) [file 10434_2023_13824_MOESM1_ESM.docx]

**SUPPLEMENTAL MATERIALS**

**Sample size calculation and statistical analysis**

***Hypothesis testing***

The detection rate of the two methods (BD-RI and ICG-RI) was compared using the chi-square test, assuming an alpha error of 5% as the index of statistical significance. The BD-RI technique was treated as gold standard with established evidence of capturing at least 97% of the SLNs. At least 260 SLNs excised were calculated to demonstrate equivalence between the two dual techniques with a 5% margin, 5% alpha error, and 80% power.

pwr.chisq.test(w=0.18, df=3, sig.level=0.05, power=0.80)

Chi squared power calculation

w (effect size) = 0.18

N (number of observations) = 256.498

df (degrees of freedom) = 3

sig.level (significance level) = 0.05

power = 0.8


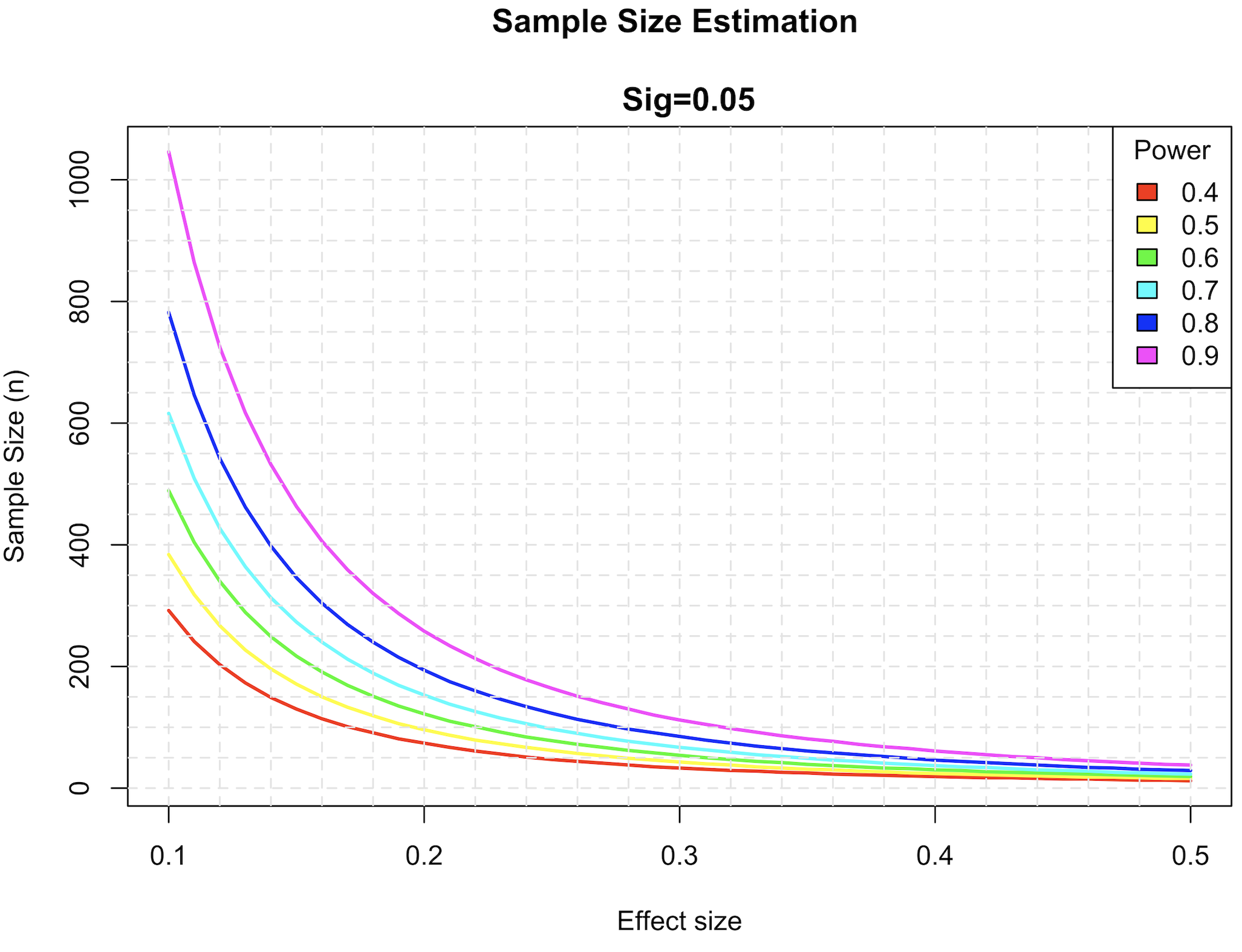


***Normality testing***

Normality testing for presentation of parametric and non-parametric data presented below. Data were evaluated for normality of distribution. A large p-value indicated that the data was normally distributed, while a low p-value indicated that it was not normally distributed.

Variables with non-normality: age at surgery, ASA grade, tumour size

Shapiro-Wilk normality test

data: SLNB$age

W = 0.98202, p-value = 0.0008091

Shapiro-Wilk normality test

data: SLNB$asa

W = 0.49636, p-value < 0.0001

Shapiro-Wilk normality test

data: SLNB$tumour_size

W = 0.86386, p-value < 0.0001

Variables with normality: BMI, surgery duration, length of hospital stay

Shapiro-Wilk normality test

data: SLNB$bmi

W = 0.77769, p-value = 0.122

Shapiro-Wilk normality test

data: SLNB$ot_duration

W = 0.72268, p-value = 0.3052

Shapiro-Wilk normality test

data: SLNB$los

W = 0.71132, p-value = 0.222
